# Visual Evoked Potentials to Monitor Myelin Cuprizone-Induced Functional Changes

**DOI:** 10.3389/fnins.2022.820155

**Published:** 2022-04-15

**Authors:** Silvia Marenna, Su-Chun Huang, Gloria Dalla Costa, Raffaele d’Isa, Valerio Castoldi, Elena Rossi, Giancarlo Comi, Letizia Leocani

**Affiliations:** ^1^Experimental Neurophysiology Unit, Institute of Experimental Neurology (INSPE), IRCCS-Scientific Institute San Raffaele, Milan, Italy; ^2^Faculty of Medicine, Vita-Salute San Raffaele University, Milan, Italy; ^3^Casa di Cura Privata del Policlinico, Milan, Italy

**Keywords:** mouse model, visual system, non-invasive visual evoked potentials, cuprizone model, demyelination, remyelination

## Abstract

The visual system is one of the most accessible routes to study the central nervous system under pathological conditions, such as in multiple sclerosis (MS). Non-invasive visual evoked potential (VEP) and optical coherence tomography (OCT) were used to assess visual function and neuroretinal thickness in C57BL/6 taking 0.2% cuprizone for 7 weeks and at 5, 8, 12, and 15 days after returning to a normal diet. VEPs were significantly delayed starting from 4 weeks on cuprizone, with progressive recovery off cuprizone, becoming significant at day 8, complete at day 15. In contrast, OCT and neurofilament staining showed no significant axonal thinning. Optic nerve histology indicated that whilst there was significant myelin loss at 7 weeks on the cuprizone diet compared with healthy mice, at 15 days off cuprizone diet demyelination was significantly less severe. The number of Iba 1^+^ cells was found increased in cuprizone mice at 7 weeks on and 15 days off cuprizone. The combined use of VEPs and OCT allowed us to characterize non-invasively, *in vivo*, the functional and structural changes associated with demyelination and remyelination in a preclinical model of MS. This approach contributes to the non-invasive study of possible effective treatments to promote remyelination in demyelinating pathologies.

## Introduction

Multiple sclerosis (MS) is a complex autoimmune disease of the central nervous system (CNS) characterized by pathological features of inflammation, demyelination, axonal transection, and neurodegeneration (Kotelnikova et al., 2017). In the CNS, oligodendrocytes generate multiple myelin layers around axons, enabling fast and efficient nerve conduction (Stadelmann et al., 2019). In MS oligodendrocytes are damaged and myelin is lost, a process called demyelination. When axons become demyelinated following oligodendrocyte cells death, their functioning is impaired and electrical conduction is reduced. The abnormalities in electrical conduction may be measured using evoked potentials (EPs) (Leocani and Comi, 2014). In particular, the visual system offers a unique opportunity to study non-invasively the effects of subclinical relapses in patients with MS over time, through the combination of functional information from visual EPs and structural information from optical coherence tomography (OCT) (Costello and Burton, 2018; Guerrieri et al., 2021). These tools were revealed to be useful in testing remyelinating treatments in clinical trials (Cadavid et al., 2017), and are feasible in pre-clinical settings (Marenna et al., 2020).

The copper-chelating agent cuprizone (bis-cyclohexanone-oxalyldihydrazone, CPZ), administered in the diet, is among the most used agents for investigating demyelination and remyelination mechanisms in the pre-clinical setting (Praet et al., 2014), including drug testing (Deshmukh et al., 2013). Cuprizone induces demyelination by reducing energy metabolism through a toxic oxidative effect on mitochondria (Gudi et al., 2009), which is reversible after returning to a normal diet (Mohamed et al., 2019). From 1960 onward, cuprizone has been established as a neurotoxin for rodents (Carlton, 1966; Matsushima and Morell, 2001; Yu et al., 2017). While initial works have described demyelination in the CNS, particularly in the corpus callosum (Skripuletz et al., 2008) and spinal cord (Herder et al., 2011), the involvement of the peripheral nervous system (PNS) of rodents has also been described (Ünsal and Özcan, 2018; Mirzaie et al., 2020). More recently, the cuprizone diet is associated with demyelination in the optic nerve (Bagchi et al., 2014; Namekata et al., 2014; Hainz et al., 2017) and functional retinal abnormalities (Namekata et al., 2014). For instance, a recent study showed a thickness reduction of the myelin sheath (g-ratio) in the optic nerve in mice kept on a cuprizone diet for 3 weeks (Hainz et al., 2017). Bagchi and coworkers showed similar results reporting a 20% myelin loss in the optic nerve of mice following 8 weeks of cuprizone diet, with noticeable loose myelin layers around the axon and paranodal abnormalities in the survived myelin (Bagchi et al., 2014). Moreover, in 2014, a study was reported on retinal function and morphology following cuprizone administration. After 12 weeks of cuprizone diet, visual function monitored *via* multifocal electroretinogram recording was found to be altered in cuprizone-treated mice, although neither retinal morphology changes nor degeneration of RGCs was observed (Namekata et al., 2014). However, to our knowledge, functional characterization of the visual system in the cuprizone model is currently lacking. More information is needed on myelin functional characterization in the optic nerve in this model, which may be useful in functional testing of remyelinating drugs.

In the present work, we used non-invasive VEPs to investigate myelin degeneration and possible axonal damage by measuring, respectively, latency, N1 component, and peak-to-peak amplitude, N1-P2, of the acquired signal (Marenna et al., 2020). The possibility offered by this technique to provide a double characterization of the visual response is particularly important since axonal demyelination and neuronal cell death are now well-recognized as fundamental characteristics of MS pathology (Frohman et al., 2008).

Additionally, as optic nerve fibers generate from retinal ganglion cells, OCT was applied in the study to monitor and detect cell death, in ganglion cells layer (GCL) and axonal loss, in retinal nerve fiber layer thickness (RNFL). Indeed, OCT technology is increasingly being used in preclinical settings, enabling cross-sectional imaging of tissue microstructure in real-time (Wang and Chen, 2014).

Thus, taking advantage of non-invasive methods (VEP and OCT), the aim of our work was to characterize alteration and restoration of the anterior visual system function in the cuprizone model. This comprehensive approach to analyze the anterior visual pathway could be exploited in studies testing new drugs for remyelination, to effectively monitor function and morphology during treatment.

## Materials and Methods

### Animals and Cuprizone Diet

A total of 8-week-old C57BL/6 male mice (Charles River; Calco, Lecco, Italy) were randomly assigned to either 7 weeks of feeding with chow containing 0.2% cuprizone (CPZ, Sigma-Aldrich) or their usual food for the same period of time. At the end of week 7, all mice were fed with a normal diet and monitored for 15 additional days. Mice were allowed 1 week of acclimatization before the start of the experiments and were housed under a controlled 12/12 h light/dark cycle, with lights on at 9:00 a.m. Tap water and food were provided *ad libitum*. This study was conducted in accordance with the animal research: reporting of *in vivo* experiments (ARRIVE) guidelines and the European Community guidelines (Directive 2010/63/EU) and approved by the San Raffaele Institutional Animal Care and Use Committee (IACUC).

### Experimental Protocol

The experimental protocol was composed of two experiments, the first with longitudinal VEPs and OCT monitoring on and off CPZ diet, the second cross-sectional was performed for histological analysis.

In Experiment 1 (longitudinal), 20 mice underwent VEP before 1:1 randomization to receive CPZ or normal diet for 7 weeks, and then repeated 1 time a week during CPZ diet (4, 5, 6, 7 weeks on CPZ diet), and after return to normal diet (5, 8, 12, 15 days off CPZ). OCT was performed after 7 weeks on and after 5 days off CPZ.

In Experiment 2 (cross-sectional), performed 6 months apart, 16 mice received a CPZ diet for 7 weeks for histological analysis. One subgroup (*n* = 8) was sacrificed after 7 weeks on the CPZ diet, while the remaining 8 mice at 15 days after return to normal diet (off CPZ). VEPs were performed before sacrifice. To decrease the number of Healthy mice involved in the experiment, histological and VEPs results of CPZ groups were compared with a unique Healthy group (about 10 weeks old; *n* = 8 mice).

### Visual Evoked Potentials

Non-invasive epidermal VEPs were recorded using a 6 mm Ø Ag/AgCl cup electrode placed on the shaved scalp over V1, contralateral to the stimulated eye and a needle electrode was inserted in the nose for reference. The cup was fixed with electro-conductive adhesive paste over one hemisphere, and once completed the recording with the first eye, it was moved to the other hemisphere, as described previously (Marenna et al., 2019). Mice were anesthetized intraperitoneally (80 mg/kg ketamine, 10 mg/kg xylazine) and an adequate level of anesthesia was verified by checking for the presence of tail-pinching reflex. Body temperature was maintained at 36.5 ± 0.5°C by a homeothermic blanket system with a rectal thermometer probe. To dilate the eyes 1% tropicamide was used, while the ophthalmic gel was applied to prevent drying. Before the procedure, each mouse was placed in a dark room and allowed to adapt to the darkness for 5 min. Then, flash stimuli (260 mJ intensity, 10 μs duration, 1 Hz frequency), was delivered with a flash photostimulator placed at 15 cm from the stimulated eye. The non-stimulated eye was covered with a black silicone band.

For each session, 3 averages of 20 EEG segments of 500 ms duration starting at the onset of each flash were recorded (Micromed System Plus Evolution, Mogliano Veneto, Italy; sampling frequency 4,096 Hz, bandpass-filter 0.16–1024, 16 bits coding, bandpass-filter 5–100 Hz, notch filter 50 Hz).

Visual evoked potentials were measured offline, marking latency and amplitude of the N1 component and peak-to-peak N1-P2 amplitude.

### Optical Coherence Tomography

After VEPs recording, mice underwent bilateral circular peripapillary scans with a Micron IV Image-Guided OCT for rodents (Phoenix Research Labs; Pleasanton, CA, United States). Mice were anesthetized intraperitoneally (80 mg/kg ketamine, 10 mg/kg xylazine). Pupils were dilated with 1% tropicamide and ophthalmic gel (2% hydroxypropylmethylcellulose) was applied frequently to the cornea to prevent dehydration and to reduce frictions between the OCT lens and the eye. Circular scans were centered at the optic nerve head and acquired from both eyes with a diameter of 1,085 μm. Every circular scan was averaged from 5 B scans (each with 1024 A-scans) with an axial resolution of 1.2 μm. The acquired images were examined for quality control as suggested in the OSCAR-IB guideline (Tewarie et al., 2012; Schippling et al., 2015). In-house automatic segmentation software written with MATLAB R2016b (Mathworks, Natick, MA, United States) was used to separate the neuronal ganglion cell complex (NGCC), as the combined RNFL, GCL, and inner plexiform layer. To minimize the influence of respiratory movements, the analysis was performed averaging three images acquired consecutively.

### Immunohistochemistry Staining

After dislocation under deep anesthesia, optic nerves were extracted at the chiasma level, fixed in 4% paraformaldehyde overnight, and then embedded in paraffin. For each nerve, four longitudinal optic nerve sections (8 μm thickness) were obtained from 8 optic nerves for Healthy and 7 for each CPZ group (2 samples were discarded for the poor quality). The number of activated microglia/macrophage cells was determined in 2 sections for each nerve by rabbit anti-ionized calcium-binding adaptor molecule-1 antibody (1:500, Iba1, Wako, Osaka, Japan). Iba1^+^ cells were counted and normalized on mm^2^. Myelin was stained using Luxol Fast Blue (LFB) in 2 sections, and quantified as a percentage of the demyelinated area on the whole optic nerve section using the following formula (Marenna et al., 2020):

*x* = (demyelination area/total optic nerve section area) * 100.

Images of the optic nerves were obtained using an Olympus BX-51 light microscope (Olympus, Japan) equipped with a digital capturing system, Leica Application Suite X (LAS X). All histological quantifications were performed with ImageJ 1.53a (National Institutes of Health, United States; available at: https://imagej.nih.gov/ij/).

**FIGURE 1:**
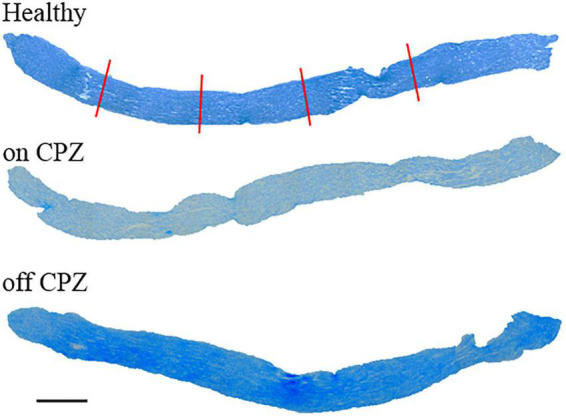
Longitudinal optic nerves were stained by Luxol Fast Blue for each group. Red lines represent the thickness measure. Scale bar, 200 μm.

Optic nerve thickness was measured in four axial segments spaced 300 μm (Figure 1), starting at least 300 μm from both extremes and covering about 1,000 μm (Hildebrand and Mohseni, 2005).

### Immunofluorescence Staining

Immunolabeling for axons was performed on separate longitudinal sections blocked and permeabilized in 10% normal goat serum, 2% bovine serum albumin, and 0.1% Triton X-100 in phosphate-buffered saline (PBS) for 30 min at room temperature. Rabbit anti-neurofilament 200 (1:500, NF200, N4142, Sigma-Aldrich) was applied on tissue sections overnight at 4°C. Then, sections were washed with PBS and incubated with Goat Anti-Rabbit IgG (H&L) AlexaFluo 488 conjugate (1:500, ab150077, Abcam). Finally, sections were washed with PBS and mounted with a 4′,6-diamidin-2-fenilindolo (DAPI) medium (ab104139, Abcam).

For quantification, three non-overlapping fields per section were captured using Olympus BX-51 fluorescent microscopy at 40× of magnification and analyzed to calculate axonal staining intensity and percent of axon-related signal with respect to the total areas using ImageJ 1.53a software.

### Statistical Analysis

For both experiments 1 and 2, VEP and OCT distribution were inspected for normality with Shapiro–Wilk test as well as visual inspection of histograms and QQ-plots, skewness and asymmetry (<2) indicated that data could be treated with parametric tests. Histological data for demyelination and Iba1^+^ cells violated normality (Shapiro–Wilk *p* < 0.05), therefore non-parametric tests were used. In Experiment 1, repeated measures ANOVA analyses were run to compare Healthy mice and cuprizone-treated mice (CPZ) over time for body weights, VEP, and OCT parameters. Following significant effects of time and/or group, *post hoc* tests and pairwise comparisons were performed using Fisher’s Least Significant Difference (LSD). Statistical significance was considered at *p* < 0.05.

In Experiment 2, Student’s *t*-test was applied to compare Healthy mice vs. 7 weeks on CPZ diet, Healthy mice vs. 15 days off CPZ diet; 7 weeks on CPZ diet vs. 15 days off CPZ diet groups for VEP, latency and amplitude, and immunofluorescence staining. Mann–Whitney *U* test was used to compare immunohistochemistry parameters, while two-way ANOVA followed by LSD *post hoc* was performed for optic nerve thickness. Statistical significance was considered at *p* < 0.05.

Statistical analyses were carried out using Statistical Package for Social Science, SPSS software (version 23.0).

## Results

### Experiment 1: Longitudinal Visual Evoked Potentials and Optical Coherence Tomography On and Off Cuprizone Diet

#### Body Weights

**FIGURE 2:**
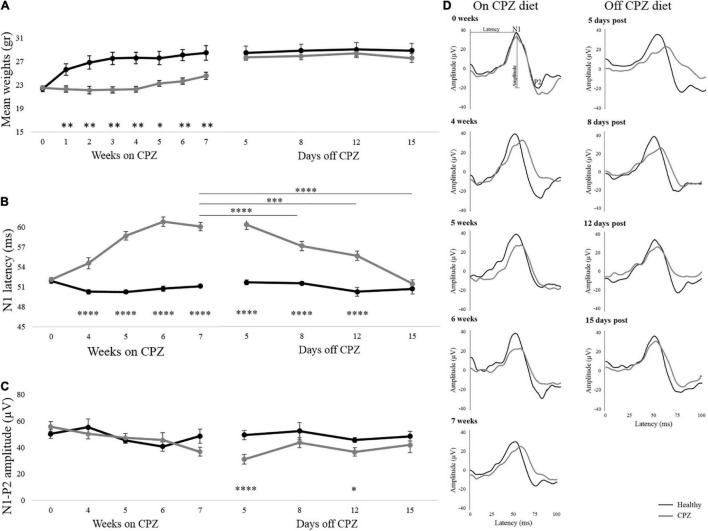
Body weights and VEPs response. **(A–C)** Mean body weights, N1 latency, and N1-P2 amplitude detected during the week on CPZ diet and days off CPZ diet. **(D)** Representative VEP waveforms of one single mouse for each group were represented during on CPZ diet (0, 4, 5, 6, and 7 weeks) and off the CPZ diet (5, 8, 12, and 15 days post). Black lines represent Healthy mice (*n* = 6 mice/12 eyes), dark gray lines represent cuprizone mice (*n* = 9 mice/18 eyes). The error bars represent the SEM. Two-way ANOVA for repeated measures followed by LSD *post hoc* test *p < 0.05; **p < 0.01; ^***^p < 0.001 and ^****^p < 0.0001.

Body weights measured during the 7 weeks on the CPZ diet are shown in Figure 2A. During this period, 4 animals in the control group and 1 in the CPZ died possibly due to mutual aggression. Cuprizone-fed animals lacked the physiological increase in body weight compared to Healthy mice (group effect: *p* = 0.001), starting from week 1 on CPZ. After returning to a normal diet (days off CPZ), body weight recovered rapidly, with no significant group effect in the interval 5–15 days (*p* = 0.439; Figure 2A and Supplementary Table 1).

#### Visual Evoked Potentials

Representative VEPs waveform recorded on and off the CPZ diet are illustrated in Figure 2D. SEM of the N1 healthy latency, reported in Figure 2B, showed high stability of this peak over time. Two-way mixed ANOVA for repeated measures, showed in Figure 2B, revealed a significant group effect on VEP latency (*p* < 0.00001), that was significantly delayed in CPZ mice already at the first time point on CPZ diet (*post hoc*, 4 weeks: *p* = 0.00008; 5 weeks: *p* < 0.00001; 6 weeks: *p* < 0.00001; 7 weeks: *p* < 0.00001; Figure 2B). During the period following interruption of CPZ diet, a significant group effect remained for VEP latency (two-way mixed ANOVA: *p* < 0.0001), with higher latencies in the CPZ group persisting up to 12 days (*post hoc*: *p* < 0.000017; Figure 2B). Effect time investigated in the CPZ group was significant (*p* < 0.0001). In particular significant N1 latency recovery was detected between 7 weeks on CPZ diet and 8 days off CPZ diet (*p* = 0.000011), 12 days off CPZ diet (*p* = 0.000023), and 15 days off CPZ diet (*p* < 0.00001; Figure 2B and Supplementary Table 2). Results for amplitudes during and after interruption of the CPZ diet showed no significant group effect, although a trend toward reduced amplitude in CPZ was observed at 7 weeks. However, VEP amplitudes showed a significant group effect (two-way mixed ANOVA: *p* = 0.003) with reduction in CPZ mice at 5 and 12 days off CPZ diet (5 days: *p* = 0.00003; 12 days: *p* = 0.02; Figure 2C).

#### Optical Coherence Tomography

**FIGURE 3:**
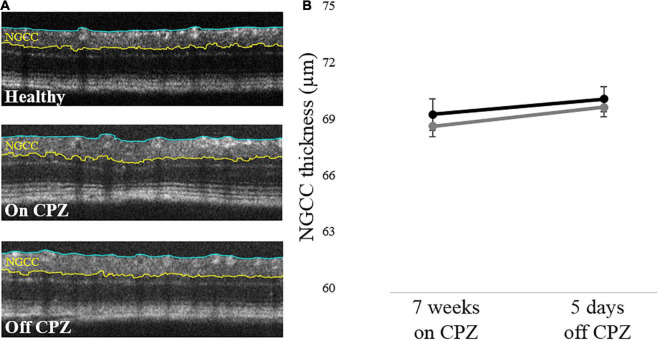
Retinal NGCC thickness of the mice with on and off CPZ diet. **(A)** Representative OCT segmentation results in Healthy, on CPZ and off CPZ mice. The thickness of NGCC was calculated as the distance between ILM (blue line) and the upper border of INL (yellow line). **(B)** The black line represents Healthy mice (*n* = 12 eyes), the dark gray line represents CPZ mice (*n* = 16 eyes). The error bars represent the SEM. Two-way mixed ANOVA showed no significant difference in the NGCC thickness between groups.

The internal limiting membrane and the upper border of the inner nuclear layer were segmented from each image, and the thickness between the two segmented lines was defined as the thickness of NGCC. Representative results of the OCT segmentation of each group are shown in Figure 3. Two-way mixed ANOVA showed no significant difference between groups (Figure 3B). Although a small effect of time was found (*p* = 0.41), *post hoc* pairwise comparisons showed no statistical significance (Supplementary Table 3).

### Experiment 2: Cross-Sectional Visual Evoked Potentials and Histology On and Off Cuprizone Diet

#### Visual Evoked Potentials

**FIGURE 4:**
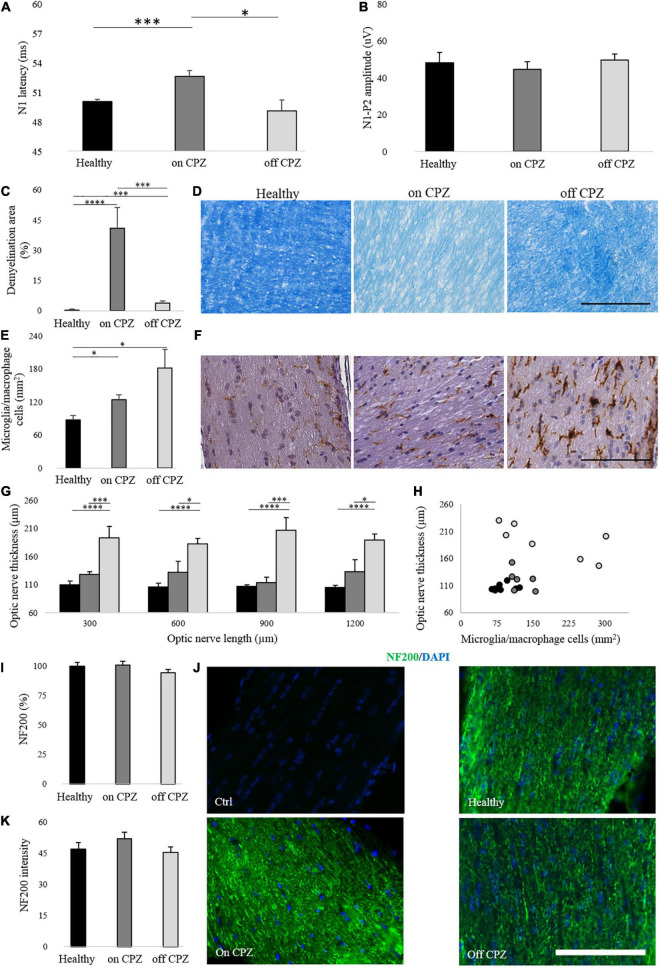
Visual evoked potentials and optic nerve histology on and off CPZ diet. **(A,B)** N1 latency and N1-P2 amplitude on CPZ and off CPZ diet. Black bars represent healthy (*n* = 16 eyes), dark gray bars represent mice on the CPZ diet (*n* = 16 eyes), light gray bars represent mice off the CPZ diet (*n* = 16 eyes). **(C)** Quantification of demyelination area (%) in optic nerves stained with LFB. **(D)** Representative optic nerve images stained with LFB. **(E)** Quantification of microglia/macrophage cells (mm^2^) in optic nerves stained with Iba 1. **(F)** Representative optic nerve images stained with Iba1^+^. **(G)** Quantification of optic nerve thickness (μm). **(H)** Correlation between microglia/macrophage cells (mm^2^) and optic nerve thickness (μm). **(I)** Quantification of NF200 (%) in optic nerve stained with NF200/DAPI. **(J)** Representative optic nerve images stained with NF200/DAPI for control of the staining and each group. **(K)** Quantification of NF200 signals intensity. Black bars/dots represent Healthy (*n* = 8 optic nerves), dark gray bars/dots represent mice on the CPZ diet (*n* = 7 optic nerves), light gray bars/dots represent mice off the CPZ diet (*n* = 7 optic nerves). **(D,F)** Images acquired by microscope in white field 40× of magnification (scale bar, 100 μm). **(J)** Images acquired by fluorescence microscopy at 40× of magnification (scale bar, 100 μm). **(A–C,E,I,K)** T-student between groups. **(G)** Two-way ANOVA followed by LSD *post hoc* *p < 0.05; **p < 0.01; ***p < 0.001 and ****p < 0.0001.

In the 7-week CPZ diet group, N1 latency was significantly delayed compared with healthy and off group (Healthy vs. on CPZ diet, *p* = 0.001; on CPZ diet vs. off CPZ diet, *p* = 0.011; Figure 4A). No significant group difference was detected for latency between healthy and off CPZ (*p* = 0.423) and for amplitudes across all groups (healthy vs. on CPZ diet, *p* = 0.589; healthy vs. off CPZ diet, *p* = 0.826; on CPZ diet vs. off CPZ diet, *p* = 0.354; Figure 4B).

In Experiment 2, N1 latency delay obtained in the group at 7 weeks on CPZ diet was significantly lower compared with the corresponding CPZ group in Experiment 1 (*p* < 0.00001; Supplementary Table 4).

#### Optic Nerve Histology

The percent of demyelination was significantly higher in both CPZ groups (on and off cuprizone; Figure 4D) compared to Healthy (Healthy vs. on CPZ diet: *p* = 0.0003; Healthy vs. off CPZ diet: *p* = 0.006) and mice on CPZ diet (*p* = 0.001) (Figure 4C). As from Iba 1^+^ cells count (Figure 4F), a significant increase in microglia/macrophage cells density was found in both CPZ groups compared to Healthy (Healthy vs. on CPZ diet: *p* = 0.029; Healthy vs. off CPZ diet: *p* = 0.04) but no significant difference was found between on and off CPZ diet groups (*p* = 0.731; Figure 4E and Supplementary Table 5). Optic nerve thickness was significantly increased in off CPZ mice compared to Healthy and on CPZ groups (Figure 4G; two-way ANOVA, *p* = 0.0003. LSD *post hoc* values in Supplementary Table 6). No significant correlation was found between optic nerve thickness and microglia/macrophage cells in any group (a trend to a negative correlation was found in the off CPZ group: ρ = –0.750, *p* = 0.052) (Figure 4H). The axonal neurofilament, NF200 antibody (Figure 4J), showed no significant groups differences in percent representation (Healthy vs. on CPZ diet: *p* = 0.954; Healthy vs. off CPZ diet: *p* = 0.427; on CPZ diet vs. off CPZ diet: *p* = 0.319; Figure 4I), as well as staining intensity (Healthy vs. on CPZ diet: *p* = 0.546; Healthy vs. off CPZ diet: *p* = 0.799; on CPZ diet vs. off CPZ diet: *p* = 0.437; Figure 4K).

## Discussion

The toxic cuprizone diet is already known to affect myelin in both CNS (Skripuletz et al., 2008; Pfeifenbring et al., 2015) and PNS (Ünsal and Özcan, 2018; Mirzaie et al., 2020). Notably, recent papers identified structural myelin alteration in the optic nerve as well (Bagchi et al., 2014; Namekata et al., 2014; Hainz et al., 2017). Currently, nothing is mentioned in the literature about the usefulness of VEPs in monitoring the evolution of demyelination and remyelination in the cuprizone model. Thus, our work aimed to investigate the value of VEPs, as a non-invasive measure to test the effects of novel remyelinating agents with the cuprizone model. Using VEPs, we were able to monitor non-invasively, through an epidermal recording, the slowing and recovery of nervous conduction along the visual pathways in the cuprizone model. We found that, compared to healthy controls, cuprizone-treated mice showed significant VEP latency delays at 4, 5, 6, and 7 weeks of diet, indicating functional impairment in the optic pathways. After the interruption of the cuprizone diet, VEPs latency gradually recovered from 12 to 15 days off cuprizone. In experimental autoimmune encephalomyelitis, VEPs delay is associated with demyelination (Marenna et al., 2020). In the present work, we found optic nerve demyelination after 7 weeks on a cuprizone diet. On the other hand, at 15 days off cuprizone, normalization of VEPs was accompanied by significantly milder demyelination compared with that after 7 weeks on cuprizone, suggesting that functional recovery corresponded to the structural restoration of myelin. Evidence in the existing literature points to cuprizone-induced demyelination within the visual pathway, specifically in the dorsal lateral geniculate nucleus whilst the optic nerve was spared (Araújo et al., 2017). Indeed, it cannot be excluded that VEP latency delays may reflect demyelination in other subcortical locations along the visual pathway. Nevertheless, the present histological results were consistent with the evidence of demyelination in the optic nerve found in previous studies (Bagchi et al., 2014; Namekata et al., 2014). Reduced myelin thickness (g-ratio) in the optic nerve in mice fed with cuprizone diet for 3 weeks has also been reported (Hainz et al., 2017). Moreover, demyelination in this model coexists with disorganization of the paranodal area with the spread of sodium channels and reduced efficiency of propagation of action potentials (Zoupi et al., 2013), thus suggesting the involvement of the optic nerve in the cuprizone model of demyelination. Further analysis of VEPs amplitudes revealed interesting results. Although no significant group differences were found during the toxic diet, cuprizone-treated mice showed a significant decrease at 5 days after returning to the normal diet, suggesting a developing axonal dysfunction. The drop in VEPs amplitude may reflect the ongoing reorganization of ion channels along the axons. Indeed, juxtaparanode abnormalities in the optic nerve of mice after 8 weeks of cuprizone diet (Bagchi et al., 2014) as well as reorganization of sodium channels in node and paranode (Zoupi et al., 2013), can be linked with conduction block by nitric oxide (Shrager and Youngman, 2017), which is also increased in the cuprizone model (Hashimoto et al., 2017).

There is evidence suggesting that different methods of cuprizone delivery could reduce the variability of toxin intake compared with food ingestion (Zheng et al., 2021). However, cuprizone-containing pelleted feed is growing in popularity, with many research groups successfully utilizing the pelleted form during diet (Toomey et al., 2021). The latency difference between the two groups on CPZ tested in the two experiments performed in different periods is likely due to differences in the cuprizone model implementation (either technical, seasonal, or both) rather than in VEPs methodology, as the two healthy groups had comparable latencies.

Another important aspect of the present findings is the association between cuprizone diet and increased density of Iba 1^+^ cells, indicating activated microglia/infiltrating macrophages, after 7 weeks on cuprizone diet, which remained elevated for the whole period after the diet interruption despite remyelination and recovery of nervous conduction, as from VEPs. There could be several explanations for this result. Microglia/macrophage cells increase during the toxic diet (Hainz et al., 2017) and the time to normalize the inflammation state is directly dependent on the CNS region (Leicaj et al., 2018). More interestingly, microglia and macrophage cells are divided into M1, pro-inflammatory, and M2, anti-inflammatory, and the balance of these two subgroups may influence disease progression and recovery (Chu et al., 2018). Thus, after returning to the normal diet, the elevated number of Iba 1^+^ cells might be consisting of an increased proportion of M2 cells, but this hypothesis is speculatory and needs further experimental exploration. Activated microglia may be beneficial to remyelination (Zhu et al., 2017), mainly contributing to the phagocytosis of myelin debris associated with demyelination (Miron, 2017). It has been reported that demyelinated axons and oligodendrocytes have a reciprocal signaling relationship in which oligodendrocytes receive cues from axons, directing myelination (Piaton et al., 2011) and the presence of oligodendrocytes increase the axonal caliber (Sánchez et al., 1996; Baumann and Pham-Dinh, 2001). Thus, we hypothesize that M2 cells phagocyte myelin debris, preparing a favorable milieu to remyelination. In addition, physiological aging is associated with increased Iba 1^+^ cells (Klein et al., 2018). As our finding of elevated Iba 1^+^ cells was from mice older than the healthy group (18 vs. 10 weeks, respectively), part of the present finding may be explained by physiological aging, although this has been found exploring mice in an older age range compared with those in our study (Klein et al., 2018).

Moving beyond typical histological analyses, not suitable for continuous monitoring of the same individuals, OCT allows the *in vivo*, non-invasive assessment of the evolution of neuroretinal damage over time. Despite abnormalities in VEPs latencies and amplitudes, we did not find axonal loss in the optic nerve as well as evident neuroretinal thinning at OCT, consistently with previously reported evidence (Namekata et al., 2014) suggesting that this model may be adequate to explore and monitor remyelination mechanisms, which cannot take place in the presence of relevant neuroaxonal loss.

The availability of a non-invasive marker of myelin function, such as VEPs, will also allow within-group statistics, improving the welfare of animals and reducing their numbers in pre-clinical studies.

In conclusion, non-invasive VEPs in the cuprizone model, characterized by reversible demyelination and conduction dysfunction, with relative neuroaxonal sparing, represent the ideal combination for testing novel remyelinating strategies.

## Data Availability Statement

The raw data supporting the conclusions of this article will be made available by the authors, without undue reservation.

## Ethics Statement

The animal study was reviewed and approved by San Raffaele Institutional Animal Care and Use Committee (IACUC).

## Author Contributions

SM contributed to the study planning, data collection, data analysis, statistical analysis, data interpretation, and manuscript writing. S-CH contributed toward data collection and data analysis, and interpretation. GD helped in statistical supervision. Rd’I helped in data collection. VC contributed toward data collection and animal handling. ER contributed toward data interpretation and manuscript writing. GC contributed toward supervision of data collection. LL contributed to the study conception and design, supervision of data collection and analysis, data interpretation, manuscript writing, and revision. All the authors contributed to revising the manuscript.

## Conflict of Interest

The authors declare that the research was conducted in the absence of any commercial or financial relationships that could be construed as a potential conflict of interest.

## Publisher’s Note

All claims expressed in this article are solely those of the authors and do not necessarily represent those of their affiliated organizations, or those of the publisher, the editors and the reviewers. Any product that may be evaluated in this article, or claim that may be made by its manufacturer, is not guaranteed or endorsed by the publisher.
