# Supplementary material for: Visual Evoked Potentials to Monitor Myelin Cuprizone-Induced Functional Changes
Source: Front Neurosci. 2022 Apr 15;16:820155. doi: 10.3389/fnins.2022.820155 (PMC9051229; doi:10.3389/fnins.2022.820155)
Supplement: Supplementary file 1 [file Table_1.DOCX]

**Supplementary material**

**Table S1: Statistics for body weights (Figure 2A)**

|  | Data | Statistical test | Statistical value (df) | | P value | Post-hoc (LSD) P value |
| --- | --- | --- | --- | --- | --- | --- |
| Body weights | Baseline to 7 weeks on CPZ diet | Two way mixed ANOVA | | F(1,13)=16.398 – group effect | p=.001 | p=.974 (Baseline)  p=.007 (1 week on)  p=.002 (2 weeks on)  p=.003 (3 weeks on)  p=.007 (4 weeks on)  p=.002 (5 weeks on)  p=.001 (6 weeks on)  p=.008 (7 weeks on) |
|  | 5 to 15 days off CPZ | Two way mixed ANOVA | | F(1,13)=.639 – group effect | p=.439 | n/a |

df: degrees of freedom

LSD: least significant difference

n/a: not applicable

CPZ: cuprizone

**Table S2: Statistics for Experiment 1: VEP latency, amplitude (Figure 2B, C)**

|  | Data | Statistical test | Statistical value (df) | P value | Post-hoc (LSD)  p value |
| --- | --- | --- | --- | --- | --- |
| VEP latency | Baseline to 7 weeks on CPZ | Two way mixed ANOVA | F(1,28)=170.593 – group effect | p<.00001 | p=.662 (Baseline)  p=.00008 (4 weeks on)  p<.00001 (5 weeks on)  p<.00001 (6 weeks on)  p<.00001 (7 weeks on) |
|  | 5 to 15 days off CPZ  7 on CPZ to 15 days off CPZ | Two way mixed ANOVA  Two way mixed ANOVA | F(1,27)=96.893 – group effect  F(2.68,42.94)=  34.233 – time effect (Greenhouse-Geisser corrected) | p<.00001  p<.00001 | p<.00001 (5 days off)  p<.00001 (8 days off)  p=.000017 (12 days off)  p=.506 (15 days off)  p=.184 (7 weeks on vs 5 days off)  p=.000011 (7 weeks on vs 8 days off)  p=.000023 (7 weeks on vs 12 days off)  p<.00001 (7 weeks on vs 15 days off) |
| VEP amplitude | Baseline to 7 weeks on CPZ | Two way mixed ANOVA | F(1,28)=.145 – group effect | p=.706 | n/a |
|  | 5 to 15 days off CPZ | Two way mixed ANOVA | F(1,27)=16.332 – group effect | p=.0003 | p=.00003 (5 days off)  p=.067 (8 days off)  p=.02 (12 days off)  p=.206 (15 days off) |

df: degrees of freedom

LSD: least significant difference

n/a: not applicable

CPZ: cuprizone

**Table S3: Statistics for Experiment 1: OCT (Figure 3)**

|  | Data | Statistical test | Statistical value (df) | P value | Post-hoc (LSD)  p value |
| --- | --- | --- | --- | --- | --- |
| NGCC thickness | 7 weeks on CPZ – 5 days off CPZ | Two way mixed ANOVA | F(1,24)=.650 – group effect  F(1,24)=4.682 – time effect | p=.428  p=.041 | n/a  p=.133 (H)  p=.225 (CPZ) |

df: degrees of freedom

LSD: least significant difference

n/a: not applicable

CPZ: cuprizone

**Table S4: Statistics for Experiment 2: VEP latency (Figure 4A, B)**

|  | Data | Statistical test | Statistical value (df) | P value |
| --- | --- | --- | --- | --- |
| VEP latency | CPZ mice exp.1 and CPZ mice exp.2 at 7 weeks on CPZ diet | Student t-test | t(32)=8.519 | p<.00001 |
|  | H vs on CPZ diet | Student t-test | t(30)=3.807 | p=.001 |
|  | H vs off CPZ diet | Student t-test | t(30)=.883 | p=.423 |
|  | On CPZ vs off CPZ diet | Student t-test | t(30)=2.772 | p=.011 |
| VEP amplitude | H vs on CPZ diet | Student t-test | t(30)=.547 | p=.589 |
|  | H vs off CPZ diet | Student t-test | t(30)=.221 | p=.826 |
|  | On CPZ vs off CPZ diet | Student t-test | t(30)=.942 | p=.354 |

df: degrees of freedom

LSD: least significant difference

n/a: not applicable

CPZ: cuprizone

**Table S5: Statistics for Experiment 2: optic nerve myelin and microglia/macrophage cells analysis (Figure 4C, E)**

|  | | Data | Statistical test | Statistical value (U) | P value |  |
| --- | --- | --- | --- | --- | --- | --- |
| Demyelination  (% of area) | | | H vs on CPZ diet | Mann-Whitney U test | U=.000 | p=.0003 |
|  | H vs off CPZ diet | Mann-Whitney U test | U=5.000 | p=.006 |  |  |
|  | On CPZ vs off CPZ diet | Mann-Whitney U test | U=.000 | p=.001 |  |  |

| Iba1^+^ cells (count/area) | H vs on CPZ diet | Mann-Whitney U test | U=7.000 | p=.029 |
| --- | --- | --- | --- | --- |
|  | H vs off CPZ diet | Mann-Whitney U test | U=10.000 | p=.04 |
|  | On CPZ vs off CPZ diet | Mann-Whitney U test | U=18.000 | p=.731 |

df: degrees of freedom

LSD: least significant difference

n/a: not applicable

CPZ: cuprizone

**Table S6: Statistics for Experiment 2: Optic nerve thickness analysis (Figure 4G)**

|  | | Data | | | Statistical test | | | Statistical value (df) | | P value | | Post-hoc (LSD)  p value |  |
| --- | --- | --- | --- | --- | --- | --- | --- | --- | --- | --- | --- | --- | --- |
| Optic nerve thickness | | Four measures of thickness for Healthy vs on CPZ diet vs off CPZ diet | | | Two way mixed ANOVA – group effect | | | F(1,16)=21.033 | | p=0.0003 | | p=0.223  (Healthy vs on CPZ diet)  p>.00001 (Healthy vs off CPZ diet)  p=0.00047  (on CPZ vs off CPZ diet) |  |
| 300 um | | | Healthy vs on CPZ diet vs off CPZ diet | | | One way ANOVA | | F(2,19)=12.558 | | p=0.0003 | p=0.306 (Healthy vs on CPZ diet)  p=0.00011 (Healthy vs off CPZ diet)  p=0.002 (on CPZ diet vs off CPZ diet) |  |  |
| 600 um | | | Healthy vs on CPZ diet vs off CPZ diet | | | One way ANOVA | | F(2,19)=9.963 | | p=0.001 | p=0.306 (Healthy vs on CPZ diet)  p=0.0003 (Healthy vs off CPZ diet)  p=0.015 (on CPZ diet vs off CPZ diet) |  |  |
| 900 um | | | Healthy vs on CPZ diet vs off CPZ diet | | | One way ANOVA | | F(2,19)=13.986 | | p=0.0003 | p=0.758 (Healthy vs on CPZ diet)  p=0.00018 (Healthy vs off CPZ diet)  p=0.001 (on CPZ diet vs off CPZ diet) |  |  |
| 1200 um | | | Healthy vs on CPZ diet vs off CPZ diet | | | One way ANOVA | | F(2,19)=10.684 | | p=001 | p=0.187 (Healthy vs on CPZ diet)  p=0.00032 (Healthy vs off CPZ diet)  p=0.013 (on CPZ diet vs off CPZ diet) |  |  |
|  | | | |  | | |  | |  | |  | |  |

CPZ: cuprizone

H: healthy

df: degrees of freedom

LSD: least significant difference

n/a: not applicable

**Table S7: Statistics for Experiment 2: Optic nerve axonal analysis (Figure 4I, M)**

|  | Data | Statistical test | Statistical value (df) | P value | Post-hoc (LSD)  p value |
| --- | --- | --- | --- | --- | --- |
| NF200  (%) | H vs on CPZ diet  H vs off CPZ diet  On CPZ vs off CPZ diet | Student t-test  Student t-test  Student t-test | t(13)=.059  t(13)=0.820  t(13)=1.039 | p=0.954  p=0.427  p=0.319 | n/a  n/a  n/a |
| NF200  intensity | H vs on CPZ diet  H vs off CPZ diet  On CPZ vs off CPZ diet | Student t-test  Student t-test  Student t-test | t(13)=0.620  t(13)=0.260  t(13)=0.805 | p=0.546  p=0.799  p=0.437 | n/a  n/a  n/a |

CPZ: cuprizone

H: healthy

df: degrees of freedom

LSD: least significant difference

n/a: not applicable
